# Precision Vestibuloplasty in the Edentulous Maxilla: Integrating Clark’s Technique With Anterior Laser-Assisted Soft Tissue Release

**DOI:** 10.7759/cureus.88690

**Published:** 2025-07-24

**Authors:** Balaji V, Pushpalatha G, Sanjay Venugopal, Dharshana Baskar, Anudeep Reddy, Sarmistha Sritam

**Affiliations:** 1 Department of Periodontology and Implantology, Sri Siddhartha Dental College and Hospital, Sri Siddhartha Academy of Higher Education, Tumkur, IND

**Keywords:** clark’s technique, edentulous maxilla, laser assisted periosteal fenestration, preprosthetic surgery, vestibuloplasty

## Abstract

This case report outlines a multidisciplinary surgical approach to vestibular deepening in a 62-year-old male with a severely resorbed edentulous maxilla and poor denture retention. To optimize the maxillary vestibular depth and prepare the arch for prosthetic rehabilitation, Clark’s technique was utilized in the bilateral posterior region, while a diode laser-assisted periosteal release and frenectomy were performed in the anterior segment. These procedures enabled effective apical repositioning of the mucosa and muscle attachments without the need for grafting, thereby enhancing the extent of immobile mucosa essential for denture stability. Periodontal dressing was applied postoperatively to maintain vestibular depth and support tissue healing. The healing phase was uneventful, with notable improvement in vestibular depth and soft tissue quality, following which the patient was referred for final prosthetic restoration. This case highlights the clinical utility of combining traditional and laser-based vestibuloplasty techniques in managing challenging edentulous cases with limited vestibular depth.

## Introduction

The rehabilitation of a fully edentulous maxilla presents significant clinical challenges, particularly when there is advanced ridge resorption and reduced vestibular depth. The depth of the oral vestibule-defined as the vertical distance from the crest of the alveolar ridge to the depth of the mucobuccal fold-is a critical determinant in the retention, stability, and function of removable prostheses. An adequate vestibular depth allows for proper denture flange extension, reduces dislodgment during mastication and speech, and facilitates effective oral hygiene maintenance by minimizing food accumulation [1,2]. Conversely, a shallow vestibule can contribute to denture instability, soft tissue irritation, and tension on the mucosa, often resulting in gingival recession, frenum pull, and chronic inflammation [3].

Vestibuloplasty refers to a group of surgical procedures aimed at deepening the vestibule by apically repositioning muscular and mucosal attachments to increase the area of attached, immobile mucosa on the alveolar ridge. This enhances the stability and retention of complete dentures, especially in cases with significant ridge atrophy [4]. The procedure may be performed using a variety of techniques, including secondary epithelialization, mucosal grafting, skin grafting, or flap advancement.

Historically, vestibuloplasty techniques have evolved over nearly a century. In 1930, Pichler and Trauner emphasized the importance of subperiosteal dissection and skin grafting from donor sites such as the hip, which laid the foundation for future soft tissue augmentation procedures [5]. Schuchardt later introduced skin grafting for the labiobuccal mandibular surface in 1952 to improve prosthetic support [6]. Obwegeser’s contributions in 1959 were pivotal in adapting vestibuloplasty techniques to the maxilla, particularly for patients with resorbed ridges and high muscle attachments [7]. His subsequent description of full-thickness floor-of-mouth lowering in 1963, including dissection of the mylohyoid and genioglossus muscles, expanded the surgical versatility of vestibuloplasty in complex cases [8].

Among the techniques developed, Clark’s vestibuloplasty remains one of the most practical for deepening the maxillary vestibule in edentulous patients. It involves partial-thickness dissection of the mucosa and apical repositioning without the need for grafting, making it a relatively straightforward and minimally invasive procedure. Stabilization is typically achieved with an acrylic surgical stent, which maintains tissue positioning during healing [9]. This technique is particularly advantageous when donor site morbidity is a concern or when surgical grafting is contraindicated.

While the widespread use of dental implants has reduced the frequency of vestibuloplasty for denture support, the procedure retains importance in specific clinical scenarios. These include patients for whom implant therapy is contraindicated due to systemic illness, financial constraints, or inadequate bone volume. Additionally, vestibuloplasty remains valuable in cases requiring soft tissue revision after ridge augmentation, or when implants are placed in non-attached mucosa requiring conversion to a keratinized tissue interface [10,11].

Careful patient selection is essential, as vestibuloplasty may be limited by systemic conditions, prior radiation therapy, or anatomical constraints. Potential complications such as postoperative pain, altered sensation (especially in the mandibular region), and cosmetic concerns must be addressed during preoperative counseling. In patients with less than 15 mm of residual ridge height, vestibuloplasty may not significantly improve ridge depth but can still provide a stable, immobile tissue bed that improves denture performance [12].

Hence, this case report aims to demonstrate the clinical advantages of integrating Clark’s vestibuloplasty with laser-assisted soft tissue management as a conservative and effective approach for enhancing vestibular depth and prosthetic function in a compromised edentulous maxilla.

## Case presentation

A 62-year-old male patient presented to the Department of Periodontology and Implantology with the chief complaint of poor retention and instability of his upper complete denture. He reported difficulty in chewing and speaking comfortably, which affected his confidence and quality of life. The patient had been completely edentulous in the maxillary arch for the past eight years and had already undergone fabrication of two maxillary dentures, both of which failed to provide satisfactory retention.

**Figure 1:**
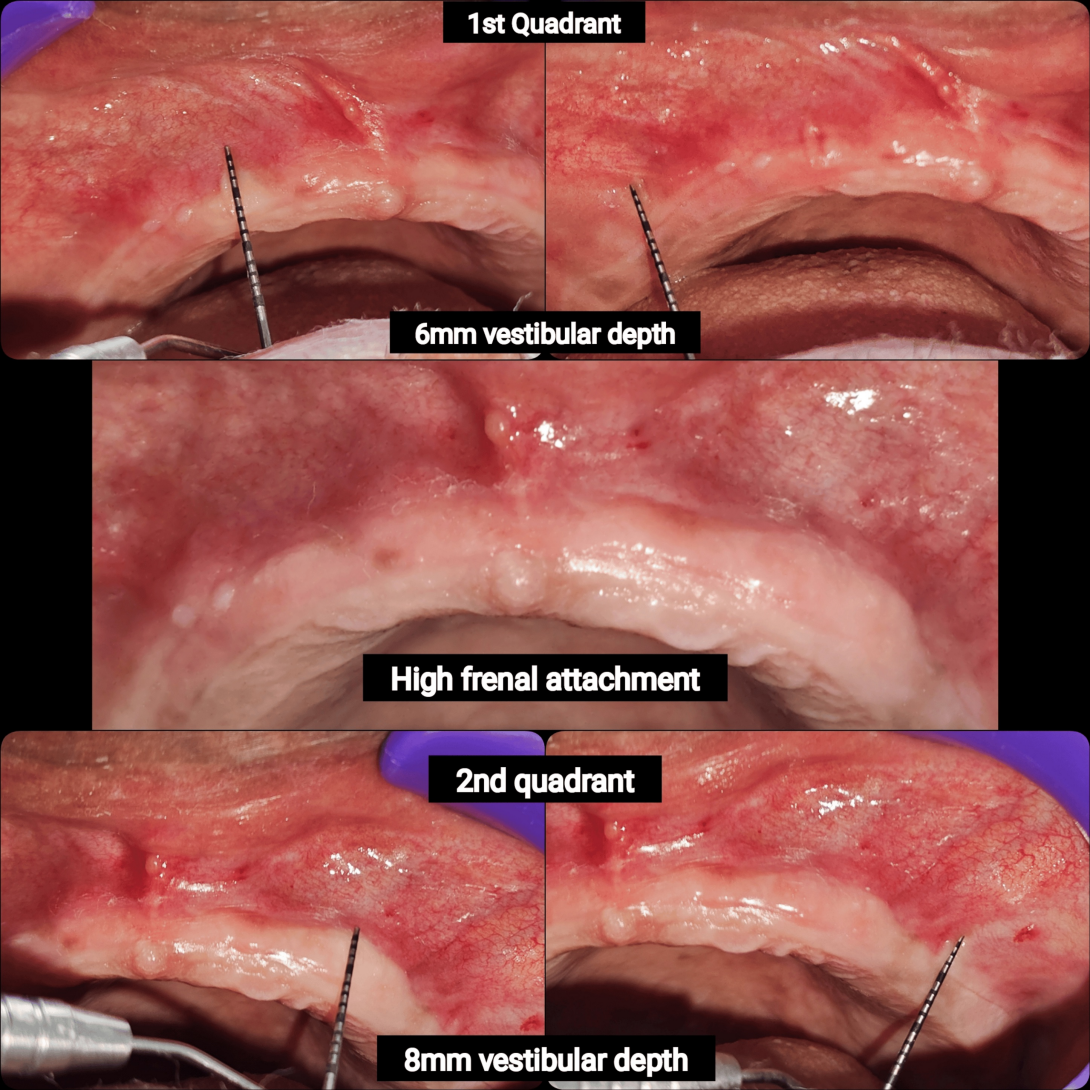
Preoperative clinical assessment of vestibular depth and labial frenal attachment The image highlights limited vestibular depth and a prominent frenum insertion, both of which can compromise prosthesis retention and necessitate vestibuloplasty.

The patient’s medical history was unremarkable, with no known systemic illnesses or history of smoking. He was not on any long-term medications and had not undergone any previous oral surgical procedures or radiation therapy. On extraoral examination, no facial abnormalities or lymphadenopathy were noted. Intraoral examination revealed a severely resorbed maxillary alveolar ridge with a narrow crest and shallow labial vestibule. The mucosa covering the ridge appeared thin and the zone of attached keratinized tissue was minimal, indicating reduced vestibular depth. Notably, a high labial frenum and close muscle insertions were present, contributing to denture dislodgement during function (Figure 1).

**Figure 2:**
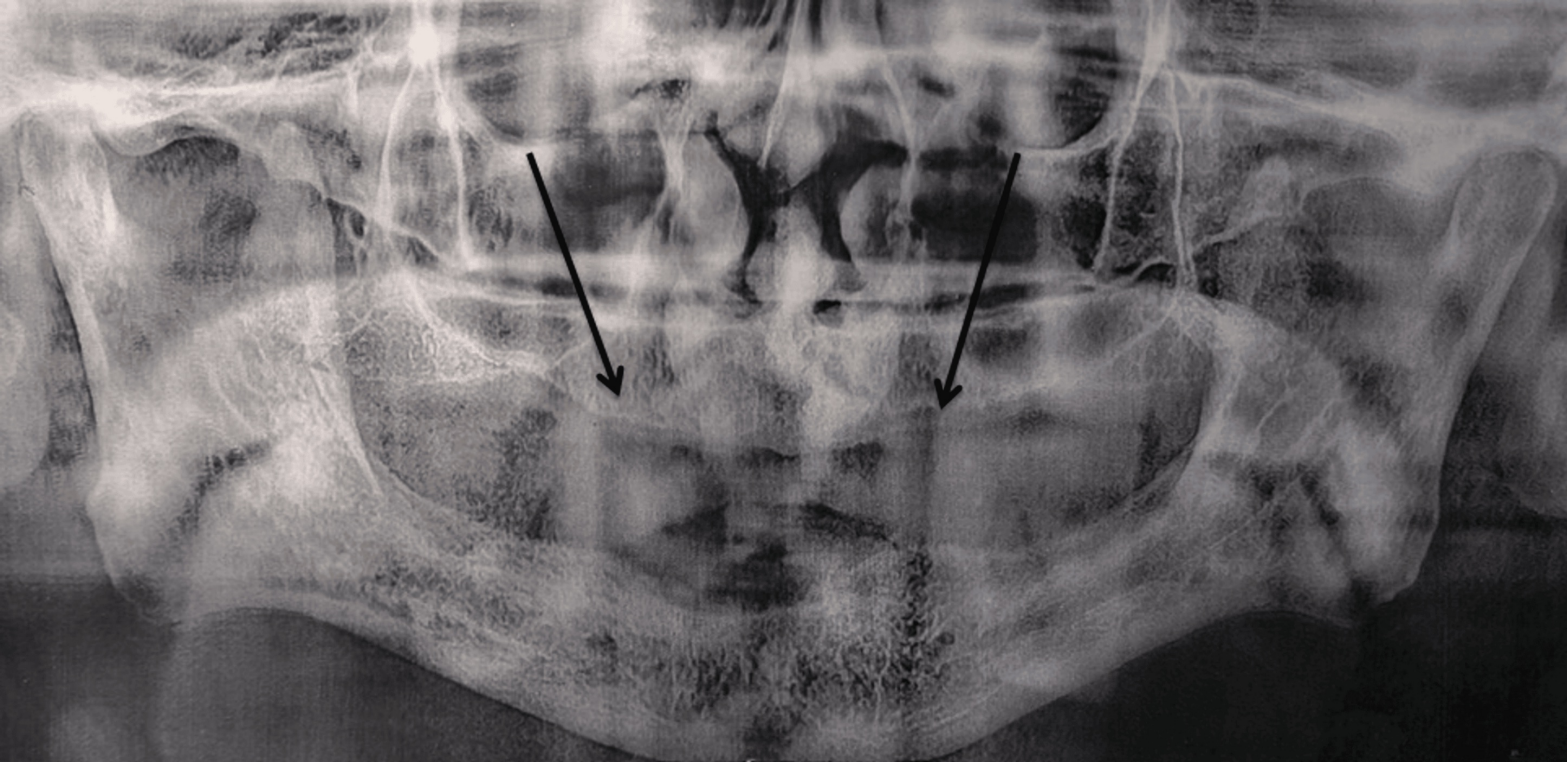
Orthopantomogram view Orthopantomogram view revealing resorbed maxillary ridge

Radiographic evaluation using an orthopantomogram showed extensive maxillary ridge resorption with no evidence of retained root fragments, bony pathology, or other anomalies (Figure 2). The limited bone volume rendered the placement of dental implants unfeasible without extensive grafting procedures, which the patient declined due to financial limitations. As a result, a conventional prosthetic approach was planned; however, the deficient vestibular depth necessitated surgical correction prior to denture fabrication.

Based on clinical and radiographic findings, a diagnosis of completely edentulous maxilla with inadequate vestibular depth was established. To enhance prosthetic retention and support, a decision was made to perform a Clark’s vestibuloplasty in the maxillary anterior and posterior regions. This technique was chosen due to its minimally invasive nature, preservation of native tissues, and ability to reposition mucosal and muscle attachments apically without the need for grafts.

The treatment plan was explained in detail to the patient, including the surgical procedure, benefits, limitations, potential complications, and postoperative care. After obtaining informed written consent, the patient was scheduled for vestibuloplasty under local anesthesia, to be followed by prosthetic rehabilitation with a new maxillary complete denture after complete mucosal healing.

Treatment

The procedure was conducted under local anesthesia using 2% lignocaine with 1:80,000 adrenaline. A combined surgical approach was followed to correct the shallow vestibule and high muscle attachments in an edentulous maxilla: Clark’s technique was utilized in the bilateral posterior maxilla, and laser-assisted periosteal fenestration was performed in the anterior maxillary sulcus and labial frenum.

**Figure 3:**
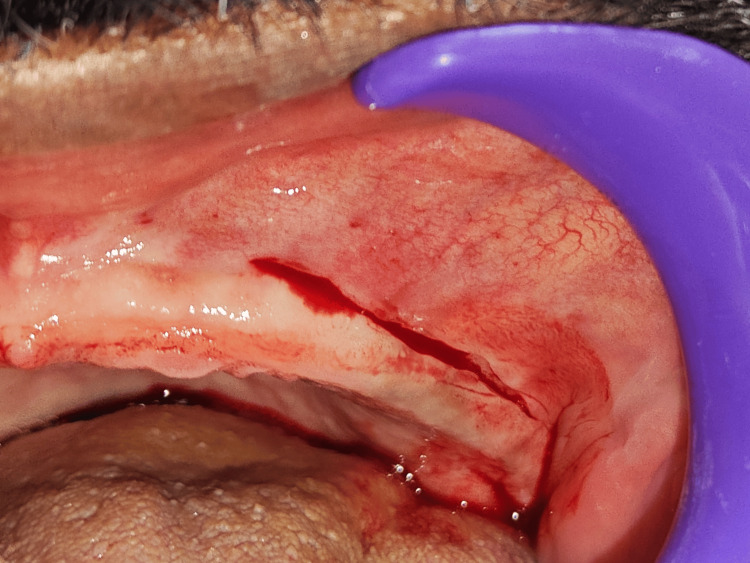
Horizontal incision given by using 15c blade A horizontal incision was made at the mucogingival junction in the posterior maxillary region using a No. 15C blade. This initial step in Clark’s vestibuloplasty technique enables apical repositioning of the mucosa to enhance vestibular depth

In the bilateral posterior maxillary vestibule, a partial-thickness horizontal incision was made approximately 2 mm superior to the mucogingival junction, beginning from the canine region and extending posteriorly to the maxillary tuberosities on each side (Figure 3).

**Figure 4:**
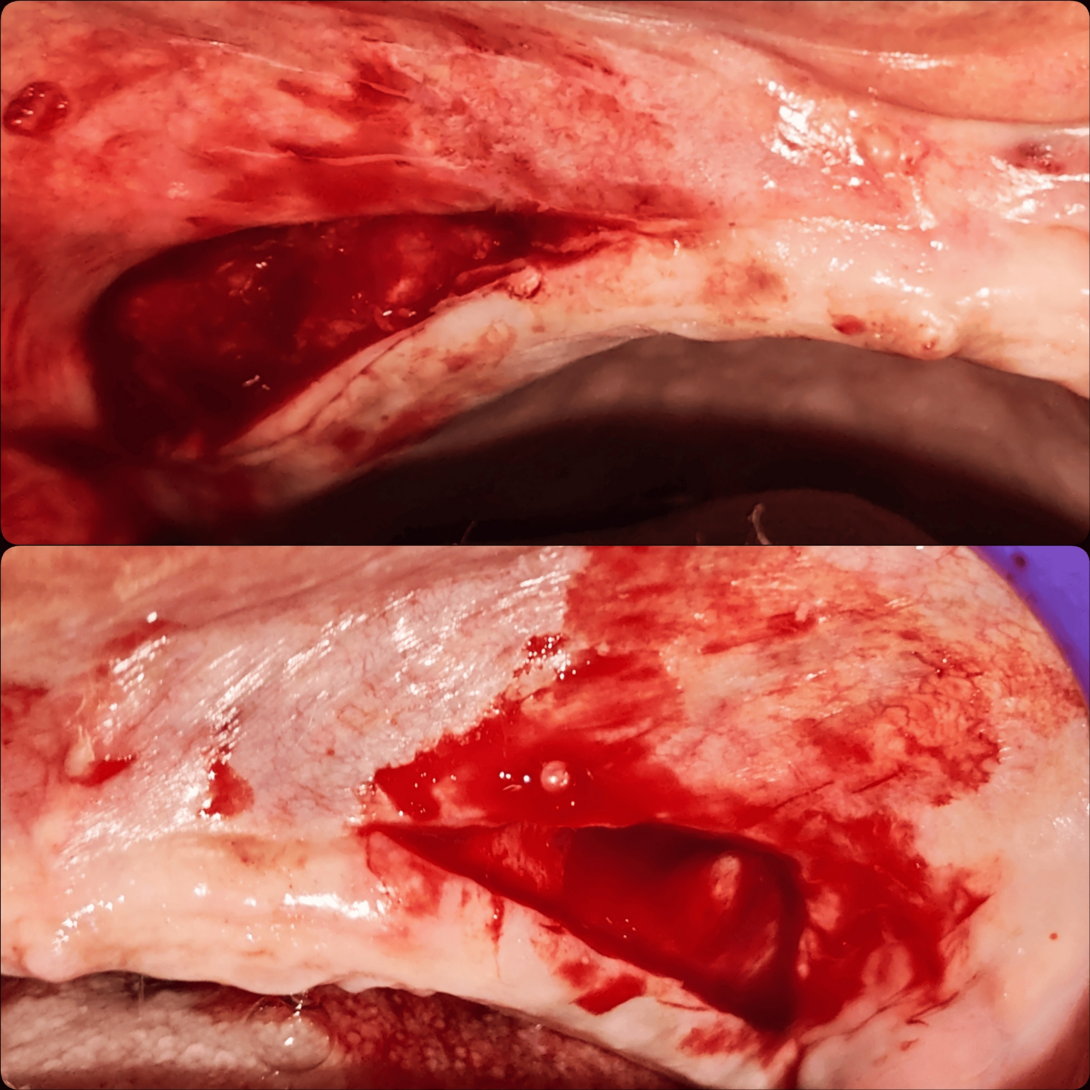
Mucosa was carefully separated from the underlying periosteum by preserving the periosteal surface This step allows for tension-free apical repositioning of the mucosal flap to deepen the vestibule while minimizing trauma to the underlying tissues

Using blunt dissection, the mucosa was carefully separated from the underlying periosteum to create a submucosal tunnel, preserving the periosteal surface (Figure 4).

This allowed for apical repositioning of the mucosa and detachment of associated muscular insertions, notably the buccinator muscle.

**Figure 5:**
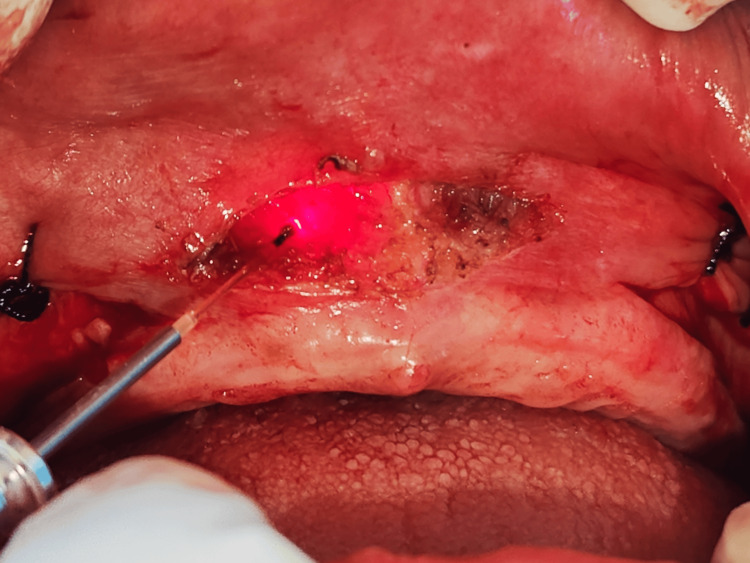
Laser-assisted labial frenectomy A labial frenectomy was performed using a diode laser to achieve precise soft tissue ablation with minimal bleeding. This was followed by controlled fenestration of the periosteum to facilitate adequate vestibular depth and reduce postoperative relapse, as part of the combined approach for vestibuloplasty.

The anterior maxillary region exhibited a high-attached labial frenum and shallow vestibular depth, contributing to prosthesis displacement. A diode laser (976 nm) with a 300 µm fiber tip was operated at 3.0 W in continuous mode for 20 seconds, delivering a total energy of 60 J was employed to perform a precise frenectomy. Fibrous attachments of the frenum were excised under local anesthesia, resulting in a bloodless surgical field (Figure 5).

**Figure 6:**
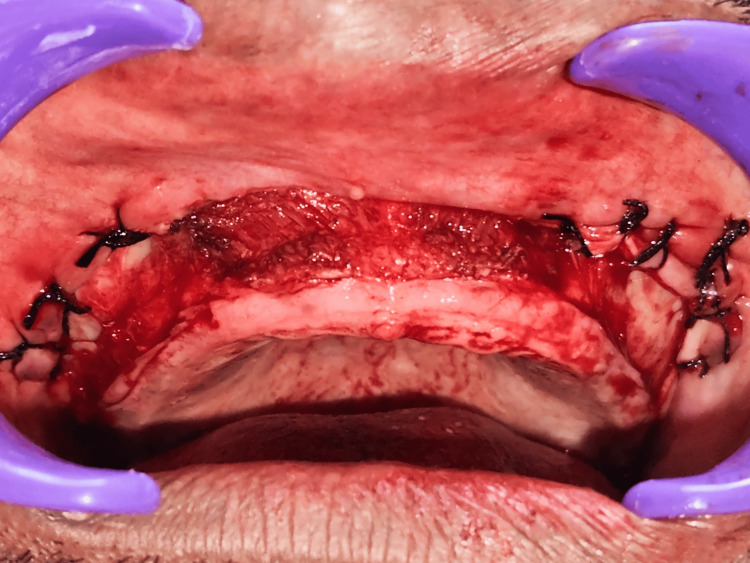
Immediate postoperative The surgical site following vestibuloplasty and laser-assisted frenectomy demonstrates well-approximated wound margins secured with interrupted sutures. Suturing was performed to stabilize the repositioned mucosa and periosteum, promoting optimal healing and maintenance of the newly created vestibular depth.

Following frenal removal, a laser-assisted linear mucosal incision was made along the midline of the anterior vestibule. Controlled fenestration of the underlying periosteum was carried out to enable the passive apical repositioning of the mucosa. The laser minimized tissue trauma, reduced postoperative edema, and eliminated the need for sutures in the anterior region. The dissected mucosa at the posterior maxilla was then sutured at its new apical position using 4-0 Mersilk to maintain the depth of the vestibule and increase the band of attached tissue. Hemostasis was achieved using sterile gauze pressure, followed by irrigation with sterile saline (Figure 6).

**Figure 7:**
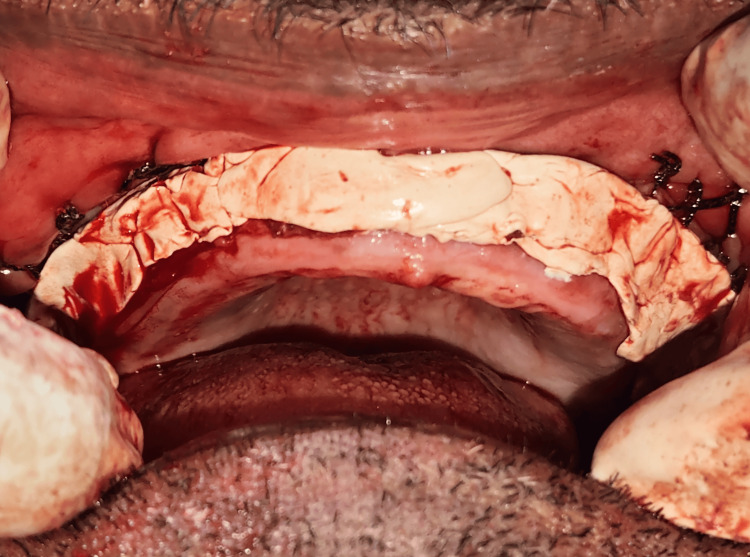
Periodontal dressing A periodontal dressing was applied to cover and protect the sutured vestibuloplasty area. The dressing helps in minimizing postoperative trauma, stabilizing the surgical site, reducing discomfort, and facilitating optimal healing by shielding the area from mechanical irritation and contamination.

After completion of both posterior and anterior vestibular modifications, a continuous layer of periodontal dressing was applied. The dressing was molded to provide support and prevent tissue rebound, aiding in the maintenance of vestibular depth during initial wound healing (Figure 7).

**Figure 8:**
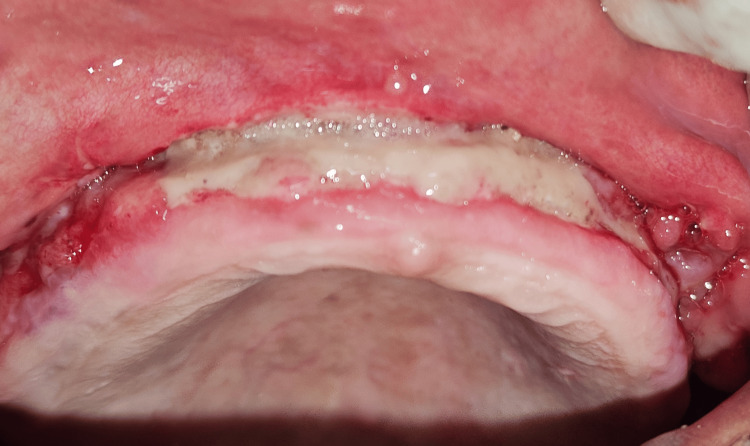
One week postoperative Clinical evaluation at one week post-surgery reveals satisfactory healing of the surgical site, with resolution of inflammation and stable repositioning of the mucosal tissues. The vestibular depth appears maintained, and no signs of infection or dehiscence are evident.

Following the procedure, the patient was given standard postoperative instructions, including avoidance of mechanical trauma and adherence to a soft diet. Oral hygiene was maintained using 0.12% chlorhexidine gluconate mouthrinse twice daily, and medications included amoxicillin 500 mg three times a day for five days and ibuprofen 400 mg three times a day as needed. The periodontal dressing was retained for seven days and removed during the first follow-up visit, revealing clean, well-adapted tissues with no signs of infection or inflammation (Figure 8).

**Figure 9:**
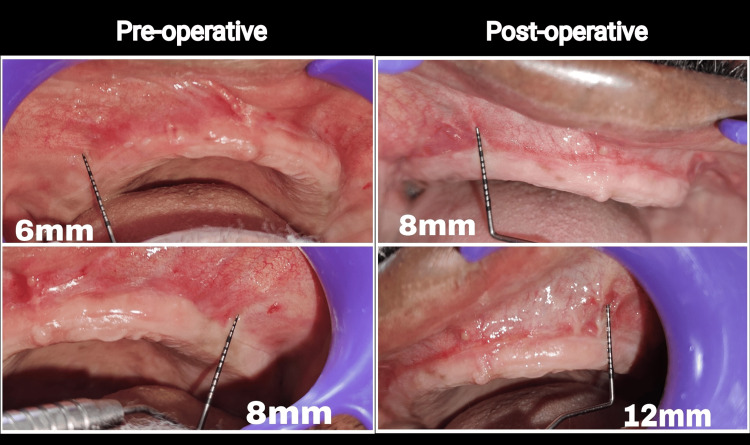
Comparison of preoperative vs postoperative The preoperative image demonstrates shallow vestibular depth with a high labial frenum attachment. The postoperative image, taken at six weeks, shows a significant increase in vestibular depth with stable mucosal positioning and complete healing. The surgical outcome reflects the effectiveness of Clark’s technique combined with laser-assisted soft tissue management.

Healing progressed uneventfully, and complete epithelialization was observed by the end of the third week. By six weeks, the vestibular depth in both anterior and posterior regions was significantly improved, with effective frenum release and no tissue rebound when compared to preoperative vestibular depth (Figure 9). The comparison of preoperative vs postoperative vestibular depth in maxilla is explained in Table 1.

**Table 1:** Preoperative vs postoperative comparison of vestibular depth in both right and left side of the maxilla This table illustrates the measured changes in vestibular depth following vestibuloplasty, showing an increase on both sides, with greater improvement observed on the left side.

Site	Preoperative Vestibular Depth (mm)	Postoperative Vestibular Depth (mm)	Increase in Depth (mm)
Right side of maxilla	6 mm	8 mm	+2 mm
Left side of maxilla	8 mm	12 mm	+4 mm

The surgical sites showed no signs of infection, dehiscence, or relapse in vestibular depth. The patient demonstrated adequate vestibular depth in both anterior and posterior maxillary regions, with favorable tissue quality suitable for prosthetic management. Upon confirmation of complete healing, the patient was referred to the Department of Prosthodontics for definitive maxillary complete denture rehabilitation.

## Discussion

Achieving adequate vestibular depth is a critical step in the preprosthetic surgical management of edentulous patients, particularly those with reduced vestibular height due to alveolar resorption. This case demonstrates a region-specific approach utilizing Clark’s technique for bilateral posterior vestibuloplasty and laser-assisted periosteal fenestration for the anterior maxilla. The dual intervention successfully deepened the vestibule, enhanced tissue stability, and prepared the arch for prosthetic rehabilitation.

Clark’s vestibuloplasty involves a mucosal flap repositioning without grafting, primarily designed to deepen the vestibule by advancing the mucosa apically while preserving periosteal attachment. When applied to the posterior maxilla, this technique minimizes donor site morbidity and facilitates healing through secondary epithelialization [13]. The procedure is particularly advantageous in patients with moderate ridge resorption, where grafting is not feasible or necessary. Clark’s method is known for its relative simplicity and predictable soft tissue outcomes when strict postoperative protocols are followed.

In the anterior maxilla, laser-assisted periosteal fenestration was selected for its precision, hemostatic control, and minimally invasive nature. Diode lasers, such as the 810-980 nm range, have been successfully used in mucogingival procedures, providing coagulation, reduced intraoperative bleeding, and better patient comfort [14]. Laser use also minimizes trauma to adjacent tissues, and the thermal effect can stimulate fibroblast activity, supporting faster tissue repair [15]. In this case, the procedure efficiently released the labial frenum and extended the anterior vestibule without sutures, promoting uncomplicated healing.

The postoperative period was uneventful, with complete epithelialization observed within three weeks. The application of Coe-Pak periodontal dressing played a pivotal role in protecting the surgical field, stabilizing the flap margins, and facilitating a moist healing environment. A single application of Coe‑Pak dressing for seven to 10 days is recommended to stabilize the vestibuloplasty site, protect healing tissues, and maintain the desired vestibular depth. If retained securely and patient hygiene is good, one application is usually sufficient, and replacement is often unnecessary unless displacement, seepage, or discomfort occurs. Previous studies have reported that laser-assisted vestibuloplasty results in less postoperative edema, reduced pain, and more rapid patient recovery compared to conventional scalpel-based techniques [16]. These findings were consistent with our observations, where the patient reported minimal discomfort and satisfactory healing throughout the follow-up period.

Both laser-assisted and conventional scalpel vestibuloplasty techniques are effective in achieving adequate vestibular depth and improving denture stability. The scalpel method is time-tested, reliable, and cost-effective, especially in settings where advanced equipment is not available. On the other hand, laser-assisted vestibuloplasty offers added advantages such as minimal bleeding, reduced postoperative discomfort, faster healing, and better patient acceptance. Each technique has its merits, and the choice may depend on patient-specific factors, clinical objectives, and operator expertise [17].

An important benefit of vestibuloplasty, particularly in the preprosthetic phase, is its role in creating a favorable foundation for prosthesis support. In this patient, the increased vestibular depth allowed for better flange extension of the final maxillary denture, ultimately improving prosthetic stability and retention. Literature suggests that successful vestibular deepening can significantly enhance the denture-bearing area, especially in severely atrophic arches [18]. Furthermore, the surgical outcome in this case eliminated high frenum pull and minimized the risk of future mucosal displacement during function.

Although implant-supported prostheses have become the gold standard in many edentulous cases, vestibuloplasty remains a valuable procedure, particularly for patients who are medically compromised, financially constrained, or unwilling to undergo implant therapy [19]. This approach is also important in settings where ridge anatomy does not allow for predictable implant placement. In such cases, conservative yet effective techniques like Clark’s vestibuloplasty combined with laser interventions offer practical and durable solutions.

Nevertheless, vestibuloplasty has limitations. Relapse of vestibular depth can occur if postoperative care is inadequate or if muscle reattachment is not prevented. Furthermore, the success of laser procedures depends on proper wavelength selection, energy settings, and operator skill. While this case did not involve the use of grafts, adjuncts such as platelet-rich fibrin (PRF) or acellular dermal matrices may further enhance long-term tissue volume and quality in more challenging scenarios [20].

## Conclusions

This case highlights the successful application of a combined surgical approach, Clark’s technique in the bilateral posterior maxilla and diode laser-assisted periosteal fenestration in the anterior region, for effective vestibular deepening in a severely resorbed edentulous maxilla. The procedure significantly enhanced vestibular depth, minimized tissue rebound, and improved the quality of the soft tissue foundation for prosthetic rehabilitation. The use of a minimally invasive laser technique ensured precise tissue management with reduced postoperative discomfort, while Coe-Pak dressing supported optimal healing. This multidisciplinary strategy underscores the importance of individualized surgical planning in preprosthetic care, especially for patients unsuitable for implant therapy, to achieve stable, functional, and retentive maxillary dentures.
